# A Case Study from the Past: “The RGC-5 vs. the 661W Cell Line: Similarities, Differences and Contradictions—Are They Really the Same?”

**DOI:** 10.3390/ijms241813801

**Published:** 2023-09-07

**Authors:** José Hurst, Gesine Attrodt, Karl-Ulrich Bartz-Schmidt, Ulrike Angelika Mau-Holzmann, Martin Stephan Spitzer, Sven Schnichels

**Affiliations:** 1 Center for Ophthalmology, University Eye Hospital Tübingen, Elfriede-Aulhorn-Str. 7, 72076 Tuebingen, Germanyu.bartz-schmidt@uni-tuebingen.de (K.-U.B.-S.); sven.schnichels@med.uni-tuebingen.de (S.S.); 2 Institute for Medical Genetics and Applied Genomics, Center for Rare Diseases, University of Tuebingen, Calwerstrasse 7, 72076 Tübingen, Germany; 3 Department of Ophthalmology, University Medical Center Hamburg-Eppendorf (UKE), Martinistraße 52, 20251 Hamburg, Germany; m.spitzer@uke.de

**Keywords:** RGC-5 & 661W, glaucoma, differentiation protocol

## Abstract

In the pursuit of identifying the underlying pathways of ocular diseases, the use of cell lines such as (retinal ganglion cell-5) RGC-5 and 661W became a valuable tool, including pathologies like retinal degeneration and glaucoma. In 2001, the establishment of the RGC-5 cell line marked a significant breakthrough in glaucoma research. Over time, however, concerns arose about the true nature of RGC-5 cells, with conflicting findings in the literature regarding their identity as retinal ganglion cells or photoreceptor-like cells. This study aimed to address the controversy surrounding the RGC-5 cell line’s origin and properties by comparing it with the 661W cell line, a known cone photoreceptor model. Both cell lines were differentiated according to two prior published redifferentiation protocols under the same conditions using 500 nM of trichostatin A (TSA) and investigated for their morphological and neuronal marker properties. The results demonstrated that both cell lines are murine, and they exhibited distinct morphological and neuronal marker properties. Notably, the RGC-5 cells showed higher expression of the neuronal marker β-III tubulin and increased Thy-1-mRNA compared with the 661W cells, providing evidence of their different properties. The findings emphasize the importance of verifying the authenticity of cell lines used in ocular research and highlight the risks of contamination and altered cell properties.

## 1. Introduction

Molecular research of ocular pathology involves studying the genetic and molecular mechanisms underlying various eye diseases and disorders. By examining the molecular changes that occur within the eye, researchers can gain a better understanding of disease progression and potential therapeutic targets. Therefore, there is a need for ex vivo or in vitro (cell) models that allow for experimental and hypothesis-driven research [1], especially since in vivo models should be further reduced because of animal welfare concerns and increasing societal pressure, as the US EPA underlined in a publication from 2019, stating a goal for research to be independent of mammalian testing by the year 2035 [2,3].

Research of retinal ganglion cell diseases requires a functioning retinal ganglion cell line to investigate the exact pathophysiological mechanisms in retinal ganglion cell diseases like glaucoma and investigate new protective approaches. For glaucoma research, it was therefore an enormous breakthrough when the successful establishment of a retinal ganglion cell line was reported in 2001 [4].

Since its establishment, the RGC-5 cell line has been widely used in the context of vision and neuronal research as it supposedly was the only existing ganglion cell line. Currently, there are about 370 articles in the literature emerging from experiments with those cells. When RGC-5 cells were initially established by the transformation of postnatal day 1 retinal cells with the psi E1A virus [4], they showed several properties of ganglion cells. The expression of neuronal markers and ganglion cell markers like Thy-1 and Brn3 and a sensitive reaction to neurotrophin withdrawal, serum deprivation and oxidative stress were confirmed by other authors [5,6].

Surprisingly, RGC-5 cells changed their characteristics over the years. Several authors proclaimed that they no longer expressed ganglion cell-specific markers and became less sensitive to glutamate excitotoxicity [7,8,9,10,11,12,13,14]. Several authors then concluded that the RGC-5 cell line dedifferentiated. Successful redifferentiation to a more neuron-specific phenotype was then achieved by treating the RGC-5 cells with either 500 nM trichostatin A [14,15,16] or 316 nM staurosporin [9,10,17,18,19]. It is known that both agents induce cell death in RGC-5 cells [15,20]. However, with both substances, the cells developed a more neuron-like phenotype, and the expression of neuronal markers increased, whereas only slight or no changes to a ganglion cell-like expression profile can be seen. 

Nevertheless, the supposed dedifferentiation also questioned the origin of the RGC-5 cell line. In 2009, van Bergen et al. [13] found out that mouse genes were expressed by RGC-5 cells, even though they were supposed to derive from rat retinas. In addition, recent reports raised concerns not only about the origin but even about the existence of RGC-5 cells [21] to an extent that the original paper was retracted. It was stated that RGC-5 cells share 95% of their gene profile with the 661W cell line, and therefore the RGC-5 cell line was most likely contaminated with the 661W cell line and, in conclusion, would be identical to 661W cells.

At this point, it was argued that it is not acceptable to further use RGC-5 cells in (the field of retinal ganglion cell) research until there is clear evidence about the true origin and the nature of these cells. Interestingly, although several journals refuse to accept any studies on RGC-5 cells because of their unknown origin, they did not retract the published papers. For example, between August 2013 and November 2015, 52 publications with research results were published based on RGC-5 cells. In 37 out of those 52 publications, these cells were used as a retinal ganglion cell model. In 11 publications, the RGC-5 cell line was described as neuro retinal precursor cells, and only 3 publications expressed some criticisms against the RGC-5 cells, while only 1 publication described a direct comparison between RGC-5 and 661W cells.

**Figure 1:**
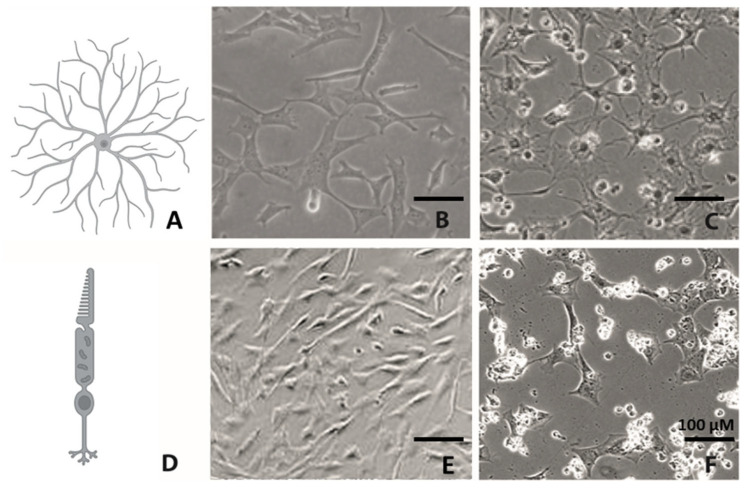
Morphology of RGC and cones and their potential cell lines. Schematic diagram of a retinal ganglion cell, modified from [26] (**A**). Phase contrast microscopy of undifferentiated RGC-5 cells in culture (**B**). RGC-5 cells after 96 h of differentiation with 500 nM TSA according to Schwechter et al. [16] (**C**). Schematic diagram of a photo receptor, modified from [27] (**D**). Untreated 661W cells in culture shortly after 24 h of cultivation (**E**). The 661W cells 96 h after differentiation with 500 nM TSA according to Schwechter et al. [16] (**F**). Scale bar = 100 µM. Created with Biorender.com.

A review of those publications which used the 661W and RGC-5 cell lines showed that they behaved quite differently in experiments [22,23,24,25]. What is more, if the cell lines are really the same, how could RGC-specific features be observed in dozens of publications [5,9,10,16,17]? The comparison of their morphologies already revealed differences between the two cell lines (Figure 1).

Are the results of the approximately 370 RGC-5 publications really useless now? Should they all be retracted? Several studies performed in vitro assays with the RGC-5 cells and later confirmed these in in vivo studies. Therefore, not all of these papers are questionable. Furthermore, in some cases, RGC-5 cells are still used for different research questions [28,29,30].

In this context, the authors’ work aims to contribute to the resolution of the contradictions surrounding RGC-5 cells by conducting a comparative analysis with the 661W cell line under controlled differentiation conditions, with the goal to reveal the differences or similarities between these two cell lines. Specifically, the authors employed two well-established redifferentiation protocols using trichostatin A (TSA) and examined various cellular properties, including morphological features and the expression of photoreceptor, neuronal and ganglion cell markers. The use of standardized redifferentiation protocols is innovative and crucial to obtaining reliable results. The two different protocols for RGC-5 differentiation with trichostatin A (TSA) are based on Schwechter et al., 2007 [16] and Wood et al., 2010 [14]. Both cell lines are incubated under the exact same conditions looking at cell amount, viability, apoptosis and especially the expression of photoreceptor, neuronal and ganglion cell markers. The HDAC inhibitor TSA is especially known to cause terminal differentiation in neuronal precursor cells [31,32]. Hence, we used these differentiation agents to differentiate the cells to their true nature, which might have been masked by several passages and the transformation of actual post-mitotic mortal cells to immortal mitotic cells.

**Figure 2:**
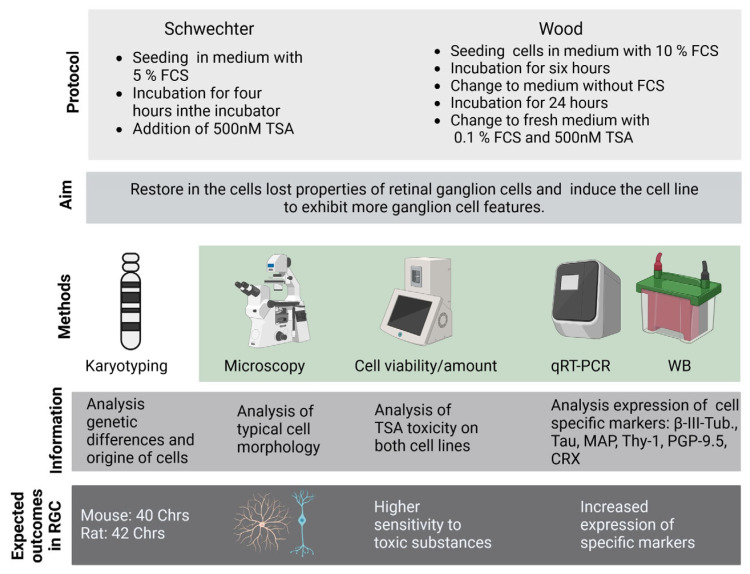
Comprehensive overview of the methods and the expected results. Abbreviations: Chr = chromosomes; FCS = fetal calf serum; qRT-PCR = quantitative real-time polymerase chain reaction; TSA = trichostatin A; and WB = Western blot. Created with BioRender.com.

Thus, this work not only helps to characterize the RGC-5 and 661W cells but is also an example of how such protocols can be used to describe cells (Figure 2).

The research gaps we intend to fill with this study are as follows:

- The origin and nature of the RGC-5 cell line: A fundamental research gap exists concerning the uncertain origin of the RGC-5 cell line. Lingering concerns regarding possible contamination with or genetic similarity to 661W cone photoreceptor cells have cast doubt on the lineage of RGC-5 cells. As such, there is a pressing need to investigate the true source and nature of the RGC-5 cell line to ensure its reliability as a retinal ganglion cell model.
- Clarifying differentiation status: Another key research gap involves determining the true differentiation status of the RGC-5 and 661W cell lines. A thorough examination is essential to ascertain whether these cell lines genuinely represent retinal ganglion cells or exhibit properties more akin to photoreceptor cells. Resolving this uncertainty is vital for accurate interpretation of experimental outcomes and targeted therapeutic studies.
- Evaluating research findings reliability: Previous studies utilizing RGC-5 cells have reported conflicting results, with some indicating ganglion cell-specific markers, while others suggested photoreceptor-like behavior. This discrepancy calls into question the reliability and validity of research findings based on RGC-5 cells. Addressing this limitation is crucial to ensure the credibility of ocular pathology studies and pave the way for robust scientific advancements.

The limitations in previous works include the following:

- Lack of standardized differentiation protocols: The absence of standardized differentiation protocols for the RGC-5 and 661W cell lines has hindered accurate comparisons of their properties. Addressing this limitation by employing rigorous and consistent differentiation procedures will enhance the reliability and interpretability of experimental results.
- Concerns about contamination and cell properties: Persistent concerns regarding potential contamination and loss of cell properties have cast doubts on the trustworthiness of research conducted using RGC-5 cells. These concerns necessitate systematic investigations to ascertain the integrity and authenticity of the cell lines under study.

## 2. Results

### 2.1. Karyotyping

Karyotyping is a powerful tool that allows scientists to study the chromosomal composition of cells and provides essential insights into genetic health, origins and differences between cells or samples.

According to the primary origin of the cell lines, 661W was expected to present murine, and RGC-5 was expected to present rat chromosomes. The normal mouse (*Mus musculus*) contains a set of 40 chromosomes, and the common rat (*Rattus norwegicus*) has 42 chromosomes. All chromosomes of *Mus musculus* (laboratory stocks, with populations of *M.m. musculus* and most natural populations of *M.m. domesticus*) are usually acrocentric, (In some populations of *M.m. domesticus* less than 40 chromosomes are found due to central fusions, or so-called Robertsonian translocations.) whereas *rattus norwegicus* has acrocentric chromosomes as well as a set of submetacentric and metacentric chromosomes.

**Figure 3:**
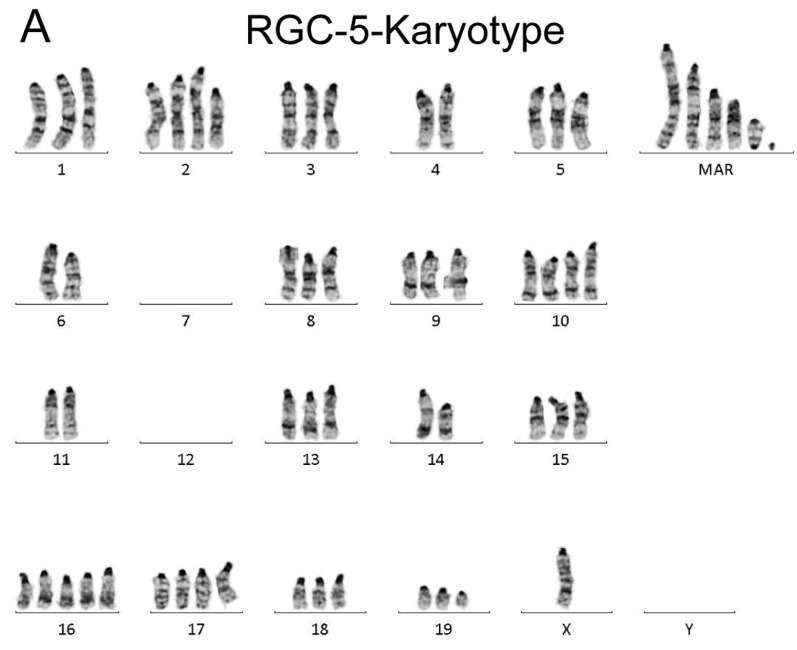

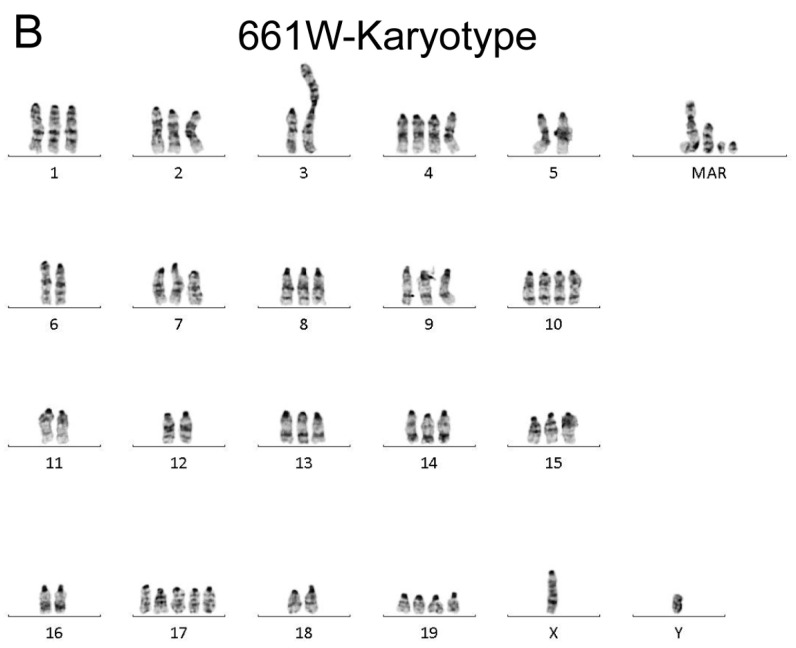
Analysis of the karyotype of RGC5- and 661W cells. Example of exclusively murine chromosomes (GTG-banded) of the RGC-5 cell line (**A**). Example of exclusively murine chromosomes (GTG-banded) of the 661W cell line (**B**).

Chromosome analysis of RGC-5 (passage 6) revealed murine chromosomes exclusively and not rat chromosomes, as expected. The chromosome number was between 55 and 59 with just one X chromosome, and a second gonosome (X or Y chromosome) was missing. Therefore, it is unclear, if the original cell line was male or female. Intercellular heterogeneity was present with a high number (4–7) of small and large marker chromosomes. Interestingly, about 50% of the cells presented with a redundant iso-dicentric chromosome (?17) which was not described previously. Both centromers were found at each end of the marker (Figure 3A). Chromosome analysis of 661W (passage 80) showed a hypertriploid murine male karyotype (60–64 chromosomes) with a variable number of small and large marker chromosomes (apparently one very large dicentric chromosome probably involving chromosome 16, which was not described before). Additionally, about 50% of the cells carried a Robertsonian translocation der(3;3), which was formerly not described in this cell line (e.g., in Krishnamoorthy et al. (2013)). Indeed, each analyzed cell showed an individual karyotype, pointing to an unstable, frequently used, manifold subcultivated cell line (Figure 3B).

Contrary to Krishnamoorthy et al., the two cell lines were quite different, having different numbers of chromosomes, a lost second gonosome in RGC-5, different numbers and types of marker chromosomes, Robertsonian translocation der(3;3) in 661W and an isodicentric marker (?17) in RGC-5 but not in 661W.

The composite karyotypes are listed below:

661W: 60~64,XY,−2,+der(3;3),−4,+5,+5,−6,−7,+10,−11,+12,+17,+17,+mar,+mar,+mar,+mar
RGC-5: 55~59,X,−4,−5,−6,+10,+11,−12,−13,−13,−14,+15,−16,+17,−18,+mar,+mar,+mar,+mar,+mar

### 2.2. Morphology Changes in RGC-5 Cells and 661W Cells Due to TSA Treatment

**Figure 4:**
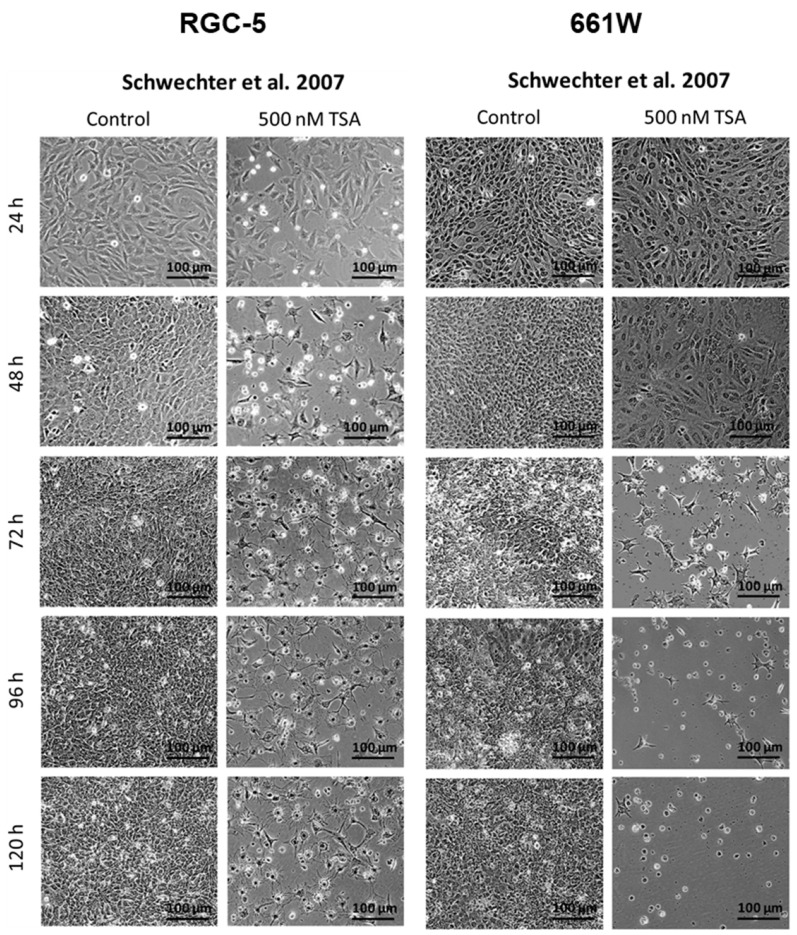
Morphology changes in TSA-treated cells according to Schwechter’s protocol [16].

**Figure 5:**
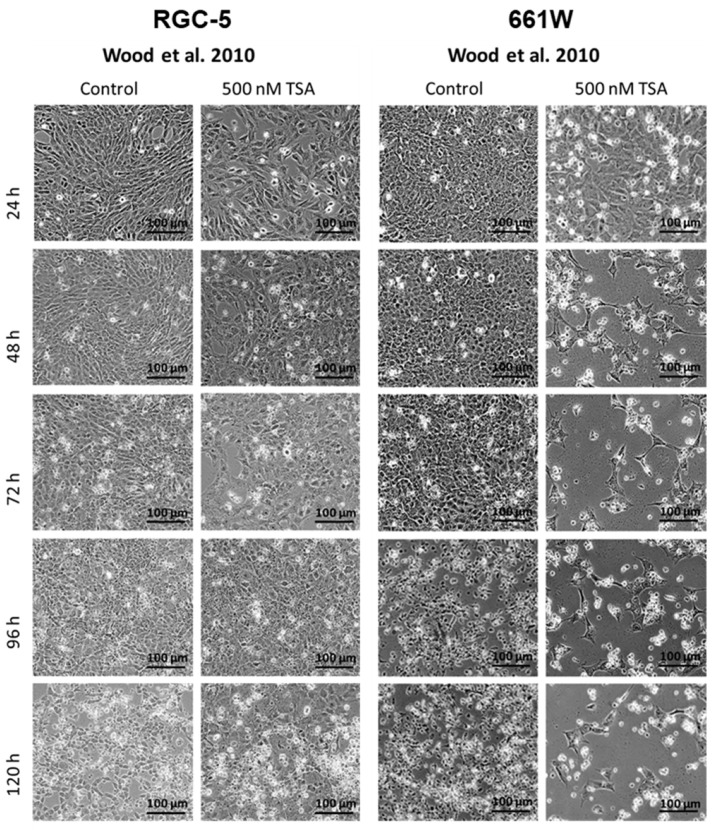
Morphology changes in TSA-treated cells according to Wood’s protocol [14].

Overall, morphological examination of cells is a fundamental method in medicine and biology that provides valuable information about cellular health or disease states, and even more importantly, it can help to characterize a certain cell type. Neuronal cells especially have a very specific morphology. To investigate the morphology of the cell lines, both cell lines where incubated with 500 nM TSA. The untreated RGC-5 and 661W cells showed a rather spindle-like shape with only few and short extensions. Under treatment with 500 nM TSA, the RGC-5 cells began to develop neurites, and their cell bodies became rounder. They changed to a ganglion cell-like phenotype, already starting after 24 h and reaching a maximum after long-term incubation of about 96 h (Figure 4 and Figure 5). The 661W cells responded differently and significantly more sensitively to treatment. A few changed their morphology slightly earlier, but they died during long-term incubation.

Representative phase contrast pictures of RGC-5 and 661W cells after 24, 48, 72, 96 and 120 h supplementation with 500 nM TSA were compared with an untreated control at 100× magnification. The RGC-5 cells showed an altered morphology under TSA treatment, already beginning after 24 h of incubation. Changing to a neuron-like phenotype, they began to develop neurites, and their cell body became rounder. They were observed to reach a maximum after long-term incubation (72 and 96 h), while the first signs of changes were seen in 661W cells after 72 h. In the longer incubation periods, no living 661W cells were detected anymore.

Dead cells lost their adhesions and were floating around in the medium (Figure 5). The 661W cells in general seemed to be more sensitive to the toxic effect of TSA than to its capability to initiate a morphology change.

Representative phase contrast pictures of RGC-5 and 661W cells after 24, 48, 72, 96 and 120 h supplementation with 500 nM TSA were compared with an untreated control at 100× magnification. The Wood redifferentiation protocol induced no morphological changes in the RGC-5 cells and a few in the 661W cells. The developed neurites were shorter, and less neurites per cell appeared. Cell death mainly occurred in both cell lines. The 661W cells were much more sensitive than the RGC-5 cells, as a massive loss of cells was already observed after 48 h. 

### 2.3. Cell Amount and Viability of RGC-5 Cells and 661W Cells during TSA Incubation

**Figure 6:**
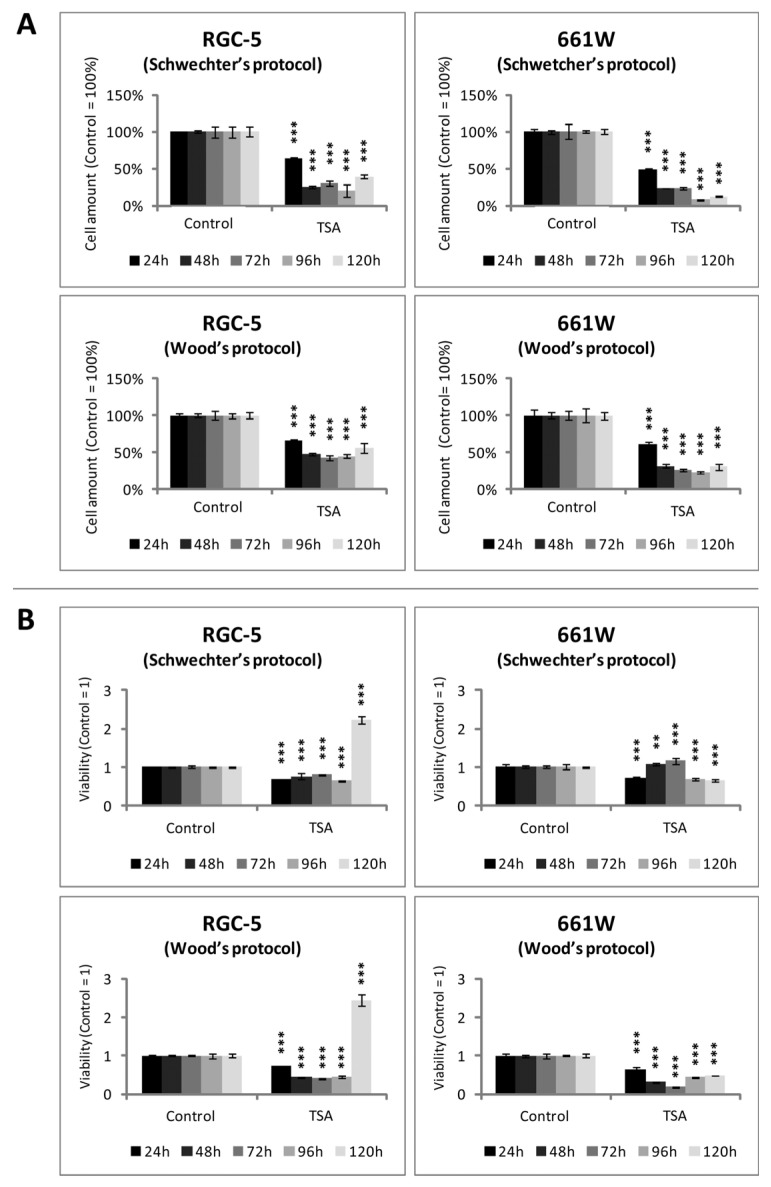
Viability and cell amount of RGC-5 and 661W cells during TSA incubation. Bar graphs represent total amount of cells (**A**) and viability (**B**) of RGC-5 cells and 661W cells after 24, 48, 72, 96 and 120 h of supplementation with 500 nM TSA, expressed as arbitrary units with control set as one and 100%, respectively (n = 6). (**A**) Crystal violet staining showed a time-dependent decrease in cell amount in TSA-treated cultures of 661W and RGC-5 cells. The smallest amount of cells resulted after 96 and 120 h of incubation with 500 nM TSA using Schwechter’s protocol. (**B**) Cell viability decreased during the early days of TSA treatment. After 120 h, RGC-5 cells showed a distinct 2.5-fold increase no matter which protocol was used. The 661W cells did not show this increase. *** = *p* ≤ 0.001. ** = *p* ≤ 0.01.

Since several publications reported a toxic effect of TSA, and we observed cell death in both populations, we tested the influence of TSA on the cell amount and viability in both cell lines. Crystal violet staining showed a time-dependent decrease in the cell amount of TSA-treated 661W and RGC-5 cells. The smallest amount of cells was found after 96 and 120 h of incubation with 500 nM TSA using Schwechter’s protocol (Figure 6A). After closer consideration of the absolute values of the crystal violet absorption results, it was obvious that in the case of Wood’s differentiation, the cell amount stayed at a constant level while treated with TSA, and only incubation according to the Schwechter protocol caused a decreased cell amount to about 30% after 120 h Aside from more or less decreasing the values of viability during the early days of TSA treatment measured with an MTS assay, the RGC-5 cells showed a distinct 2.5-fold increase after 120 h (*p* < 0.001) no matter which protocol was used for differentiation. The 661W cells did not show this increase. Using the Wood protocol, the 661W cells were always less viable than the untreated control, and upon undergoing Schwechter’s treatment, the values only increased a little after 48 and 72 h (Figure 6B).

### 2.4. The Effect of TSA on Apoptosis in RGC-5 Cells and 661W Cells

**Figure 7:**
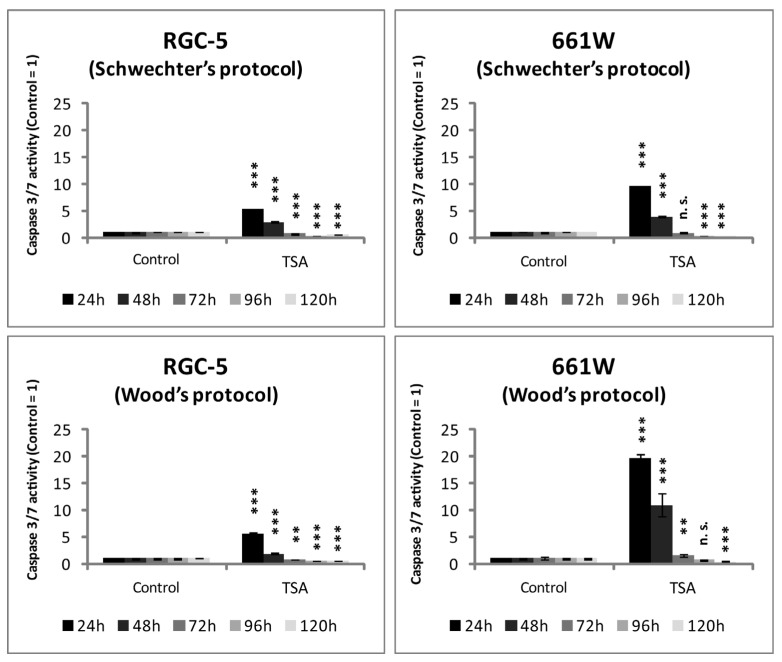
Apoptosis in RGC-5 cells and 661W cells due to TSA treatment. Bar graphs demonstrate caspase activity measured by caspase 3/7 assay as arbitrary units with control set as one (n = 6). *** = *p* ≤ 0.001. ** = *p* ≤ 0.01. Here, n.s. = not significantly different from control.

Subsequently, it should be verified whether the observed cell death was due to apoptosis. Therefore, caspase activity was measured. Both cell lines showed increased caspase activity during the first days of TSA incubation, but when treated longer than 72 h, activity decreased compared with the control. Caspase activity became very low in the RGC-5 cells, especially after 96 h of incubation using Schwechter’s protocol (21% of control, *p* < 0.0001), whereas it increased about 20-fold in the 661W cells after 24 h using Wood’s protocol (*p* < 0.0001). The 661W cells reacted more sensitively to the apoptotic stimuli of TSA, with a more distinct increase in caspase activity and a less obvious decrease in apoptosis (Figure 7).

The caspase activity increased during the first days of incubation when treated with 500 nM TSA, especially for the 661W cells. However, after long-term incubation, the caspase activity of the then differentiated cells was lower than that in the control cells. In general, the 661W cells reacted more sensitively to the apoptotic effect of TSA.

### 2.5. β-III Tubulin Expression in RGC-5 Cells and 661W Cells Caused by TSA Treatment

**Figure 8:**
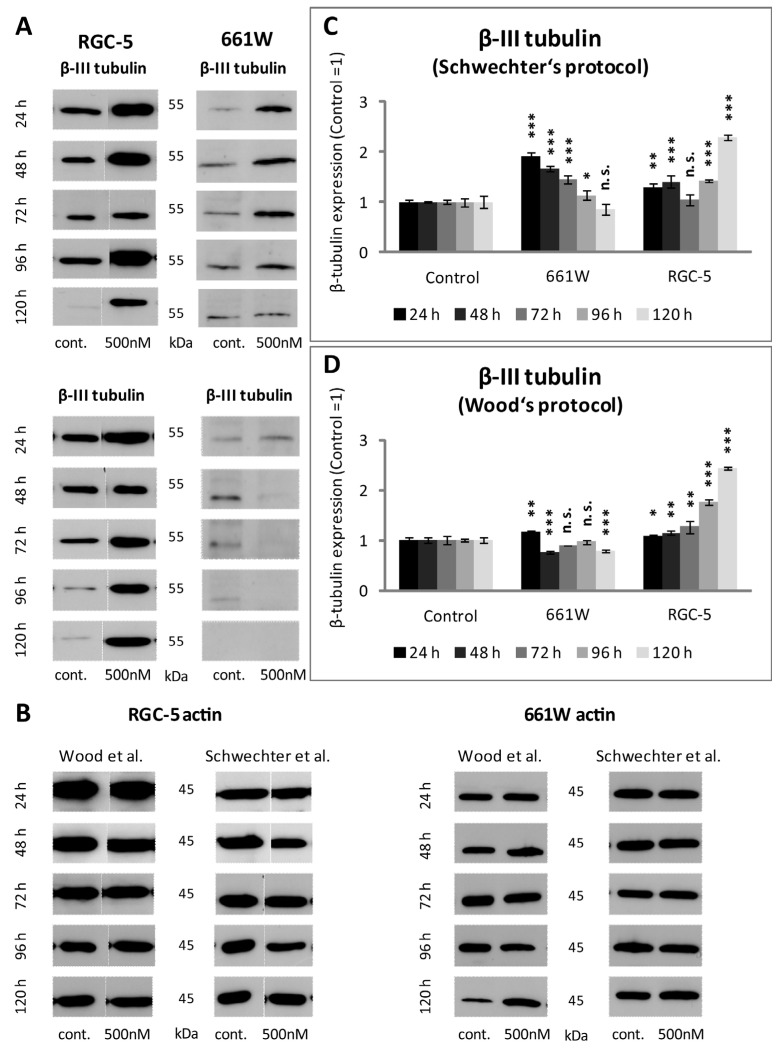
β-III tubulin expression detected via Western blotting in RGC-5 and 661W cells after TSA. (**A**) Representative pictures of β-III tubulin levels in RGC-5 and 661W cells using Schwechter’s protocol (above) and Wood’s protocol (below) after 24 h, 48, 72, 96 and 120 h. (**B**) Representative blots of reference protein actin are shown (**C**) Bar graphs representing β-III tubulin levels measured by Western blotting after 24, 48, 72, 96 and 120 h under Schwechter’s protocol. After 24 h, the RGC-5 cells already revealed a significant increase in β-III tubulin expression, with the highest expression (2.4-fold) after 120 h (*p* < 0.0001). When the 661W cells were treated according to Schwechter’s protocol, an increase in β-III tubulin expression could be observed during the first days of incubation, but after 72 h, the values dropped again. (**D**) Quantification of β-III tubulin expression of RGC-5 and 661W cells when treated according to Wood’s protocol. The RGC-5 cells reacted the same as under Schwechter’s protocol, with a significant increase in β-III tubulin which augmented over the time, whereas the 661W cells expressed slightly more β-III tubulin after 24 and 48 h, but then the amount dropped again. Control set as one (n = 4). *** = *p* ≤ 0.001. ** = *p* ≤ 0.01. * = *p* ≤ 0.05. Here, n.s. = not significantly different from control [14,16].

**Figure 9:**
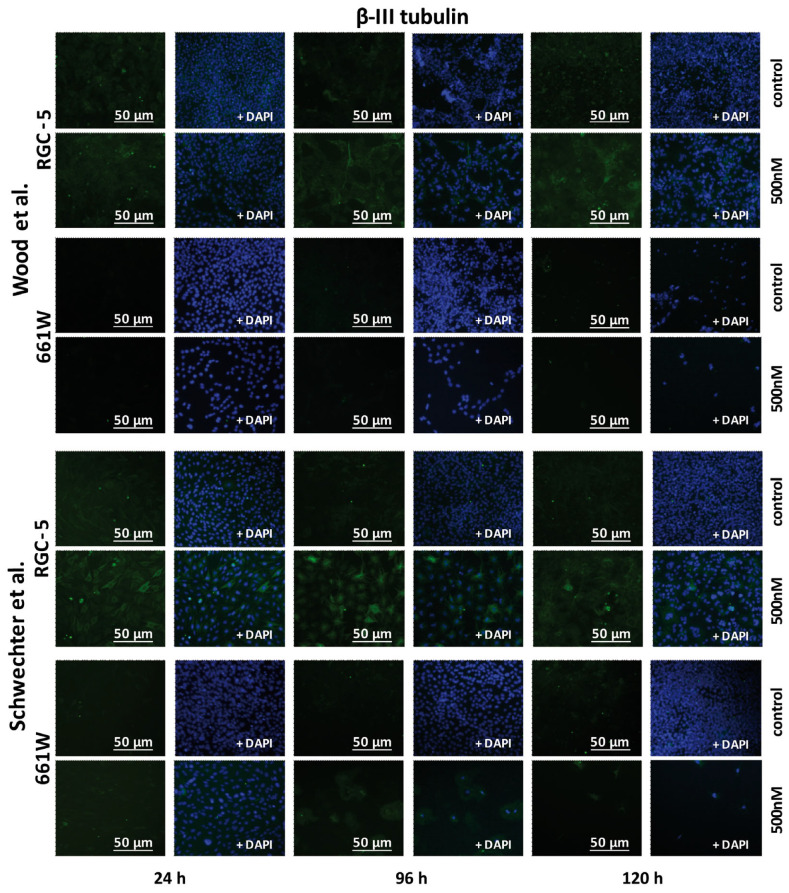
β-III tubulin immunostaining of RGC-5 cells and 661W cells [14,16].

Since β-III tubulin is a well-known neural marker, its expression was verified in both cell lines after TSA treatment. The expression of β-III tubulin should be significantly higher in ganglion cells than in photoreceptor cells. The Western blot results and immunostaining showed higher levels of expression for ß-tubulin in the RGC-5 cells than in the 661W cells. Quantification exposed a significant increase in β-III tubulin expression in the RGC-5 cells when treated with 500 nM TSA for longer than 72 h. The highest expression was detected via Western blot analysis after 120 h of incubation using Wood’s protocol, with the differentiated RGC-5 cells displaying a 2.4-fold higher expression than the untreated control (*p* < 0.0001). The 661W cells did not react the same way. When treated with TSA, they expressed slightly more β-III tubulin after 24 and 48 h but did not show an increase after long-term incubation (Figure 8 and Figure 9).

Furthermore, the expression of other neuronal markers, such as immunostained tau and MAP-2, were also examined to support the β-III tubulin results. However, there were no significant differences within the two cell lines after treatment with TSA. TSA led to an increase in MAP-2 expression in the 661W and RGC-5 cells, especially after long-term incubation. The observed effect was more pronounced when the Schwechter protocol was used. The expression of tau increased as early as after 24 h. However, there was no further increase when the cells were incubated longer.

Representative fluorescent images of β-III tubulin in RGC-5 cells and 661W cells after 24, 96 and 120 h of supplementation with 500 nM TSA were compared with an untreated control at 200× magnification. When treated with 500 nM of TSA, the RGC-5 cells showed increased β-III tubulin expression, which already started after 24 h and reached a maximum after 96 and 120 h. In the case of the 661W cells, the expression did not change significantly due to TSA treatment when incubated according to Wood’s protocol. They hardly showed any expression. When treated according to Schwechter’s protocol, a slight enhancement in β-III tubulin expression was marked after short-term incubation, but it did not increase after long-term incubation.

### 2.6. Relative Expression of Thy-1, PGP9.5, CRX- and GFAP-mRNA in RGC-5 and 661W Cells after TSA Incubation

**Figure 10:**
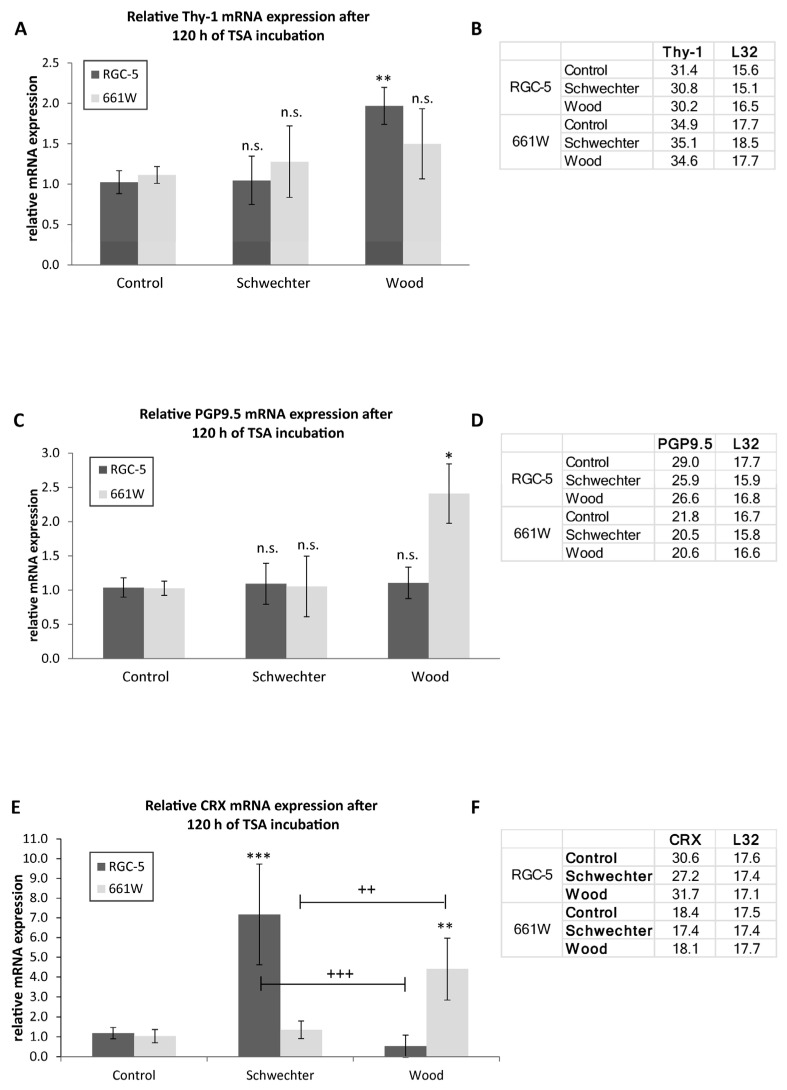
Detection of Thy-1, PGP9.5 and CRX mRNA expression. Relative Thy-1-mRNA expression in RGC-5 and 661W cells (**A**). Bar graphs picture relative mRNA expression of Thy-1 in RGC-5 and 661W cells incubated with 500 nM of TSA for 120 h as arbitrary units, with control set as one (n = 4). RGC-5 cells doubled the relative expression of Thy-1-mRNA after 120 h of TSA treatment according to Wood’s protocol, while when using Schwechter’s protocol, no difference from the control cells was observed. Thy-1 mRNA expression in 661W cells did significantly increase when treated with TSA, no matter which protocol was used. (**B**) The Thy-1 mRNA signal appeared at a CT of 30–31 in the RGC-5 cells. In the 661W cells, Thy-1 signals were later detected at a CT of 34–35. Since the same shift was observed in the housekeeping gene L32, no differences in the Thy-1 mRNA between the two cell line amounts were recordable. Relative PGP9.5 mRNA expression in RGC-5 and 661W cells (**C**). Bar graphs represent relative mRNA expression of PGP9.5 mRNA compared with control in RGC-5 and 661W cells incubated with 500 nM of TSA for 120 h as arbitrary units (n = 4). The 661W cells showed PGP9.5 mRNA expression two times higher after 120 h of TSA treatment according to Wood’s protocol, while no difference with the control cells was observed when using Schwechter’s protocol. Expression of PGP9.5 mRNA in RGC-5 cells did not significantly increase when treated with TSA. (**D**) The average PGP9.5 CT values were lower in the 661W cells (20–22) than in the RGC-5 cells, where the signal appeared at a CT of 25–27. The CT values for the housekeeping gene L32 were comparable in both cell lines, being between 15 and 17. This indicates a higher amount of PGP9.5 mRNA in the 661W cells. Relative CRX mRNA expression in RGC-5 and 661W cells (**E**). Bar graphs represent relative mRNA expression of CRX mRNA compared with control in RGC-5 and 661W cells incubated with 500 nM of TSA for 120 h as arbitrary units (n = 4). (**F**) The average CRX CT values were lower in the 661W cells (20–22) than in the RGC-5 cells, where the signal appeared at a CT of 25–27. The CT values for the housekeeping gene L32 were comparable in both cell lines, being between 15 and 17. * indicating differences to control group; + indicating differences between the two protocols. *** = *p* ≤ 0.001. ** = *p* ≤ 0.01. * = *p* ≤ 0.05. ^+++^ = *p* ≤ 0.001. ^++^ = *p* ≤ 0.01. Here, n.s. = not significantly different from control.

As the protein expression of neural markers revealed significant difference between the two cell lines, the mRNA expression of the ganglion cell marker Thy-1, PGP9.5 and the glia cell marker GFAP were checked. Additionally, the mRNA expression of the CRX gene, which encodes the cone-rod homeobox protein, was analyzed. After incubating the RGC-5 and 661W cells with 500 nM according to Schwechter’s protocol, no significant change in Thy-1 mRNA expression could be noticed. By using Wood’s protocol for TSA differentiation with 500 nM over 120 h, the relative Thy-1 mRNA expression increased twofold (*p* = 0.0028). In the case of the 661W cell line, none of the protocols changed the relative Thy-1 mRNA expression (Figure 10A). Especially notable is that the CT values of qRT-PCR showed a much earlier appearance of the Thy-1 mRNA in the RGC-5 cells than in the 661W cells (Figure 10A). (The RGC-5 cells were at a mean CT value of 30, and the 661W cells were at a mean CT value of 35.) In contrast, the mRNA expression of PGP9.5 increased significantly more than twofold in the 661W cells after using Wood’s protocol. No change in PGP9.5 expression in the RGC-5 cells with any of the protocols was observed (Figure 10B). In addition, lower CT values for PGP9.5 were detected in the 661W cells (mean CT value of 20) compared with the RGC-5 cells (mean CT value of 25), indicating a higher amount of PGP9.5 mRNA expression in general (Figure 10B).

The CRX mRNA expression increased by 4.4-fold in the 661W cells treated according to Wood’s protocol. This change in expression differed significantly from the control group and Schwechter’s protocol (*p* < 0.01) (Figure 10C). In contrast, Schwechter’s protocol resulted in a sevenfold increase in CRX mRNA in the RGC-5 cells, which significantly differed from the control (*p* < 0.001) and Wood’s protocol (*p* < 0.01). The mean CT values for CRX were 35 for the 661W cells and RGC-5 cells, whereas the mean was around 30 in the control group (Figure 10C).

The analysis of GFAP mRNA expression also revealed some differences between the two cell lines. The CT values for the RGC-5 cells were around 35.5–37, and for the 661W cells, they were above 38.5, which indicates that there was no rat GFAP mRNA detectable.

## 3. Discussion

The most important finding of the present study is that both the RGC-5 and 661W cell lines are not the same. Chromosome analysis proved a murine origin for both. In addition, they did not react the same way when treated with 500 nM TSA. While long-term TSA incubation seemed to differentiate the RGC-5 cells, the 661W cells reacted rather sensitively to the toxic effects of TSA (Figure 7 and Figure 8). Analysis of various markers (Figure 2) for the protein and mRNA levels revealed significant differences between the two cell lines (Table 1).

RGCs show several properties distinguishing them from other retinal cells. They react extremely sensitively to glutamate-induced excitotoxicity [33,34], are dependent on trophic factors [5,10,35] and undergo cell death when exposed to oxidative stress, bright light or hydrostatic pressure [11,12,36,37]. Typically, RGCs express Thy-1 and markers of the Brn3 family [38,39] and are post-mitotic.

When the RGC-5 cell line was established, it tested positive for Thy-1, showed neuronal markers like NMDA receptors and expressed neurotrophic factors as well as the corresponding receptors, while no other retinal cell markers were observed [4]. The neuronal expression profile and the ganglion cell properties were confirmed by several subsequent studies [5,6]. However, other authors did not observe sensitivity to glutamate or neurotrophin withdrawal anymore later, and the cells have tested negative for Thy-1 [7,8,9,10,11,12,13,14]. Ever since van Bergen et al. found out that RGC-5 cells are of mouse origin rather than rat origin [13] and cone opsins where detected in the cell line [14,40], the origin of the RGC-5 cell line has been most unclear. The two discussed possibilities are if vision research has to face a certain dedifferentiation of the ganglion cell line or contamination with 661W photoreceptor cells [21]. It is indeed possible that the RGC-5 cell line was contaminated with 661W cone photoreceptor cells. The 661W cells were used in the laboratory at the same time when the RGC-5 clone was isolated from rat retinas and transformed with the psi 2E1A virus. Unfortunately, some identity tests to prove the origin of RGC-5 cells have not been performed before spreading the cell line to other laboratories. One easy test would have been to check the RGC-5 genome for the transformation vector or rat-specific genes [4,21]. This suspicion is confirmed by the finding of mouse-specific genes in RGC-5 cells and the fact that the cell line is missing its transformation antigen while it is to 95% genetically identical to the 661W cone cell line [21]. The most serious argument here is that both cell lines obviously contain murine cells, as shown by karyotyping [13,21].

### 3.1. Characteristics of the RGC-5 Cells in the Present Study

As stated by Al-Ubaidi, RGC-5 cells are not exactly 661W cells [41]. His findings are in agreement with several studies conducted by us and others [14,23,25,42,43]. We therefore tested the successful differentiation protocols from Schwechter et al. and Wood et al. in parallel on 661W and RGC-5 cells to solve the mystery of if they are really the same.

First of all, the RGC-5 cells showed some expression of neuronal markers, as observed in the real-time PCR and Western blot experiments in untreated conditions. Under TSA incubation, the RGC-5 cells developed a distinct neuron-like phenotype, and the expression of neuronal markers increased. A higher expression of β-III tubulin in particular was observed. This is in accordance with previous results [14,15,35]. Using the Wood protocol, even Thy-1 mRNA increased after 120 h of incubation with 500 nM TSA, which indeed suggests a ganglion cell origin. Apart from this, the glia cell marker GFAP tested negative in qRT-PCR. As such, there is no glia cell origin of these cells. This is in agreement with another study’s results [14].

The upregulation of CRX mRNA after Schwechter’s protocol also indicates a different origin of the cells. The CRX protein encoded by this gene is a photoreceptor-specific transcription factor which plays a role in the differentiation and maintenance of photoreceptor cells [44,45,46]. Contradictory results for RGC-5 cells have been reported before. Therefore, Wood et al. performed a systematic analysis of RGC-5 cells to determine which RGC or neuronal markers are expressed after treatment with staurosporine, TSA or succinyl-concanavalin A. They postulated that neither treated nor untreated RGC-5 cells express any specific RGC marker, mRNAs or proteins like Brn-3, neurofilaments, Thy-1 or glial fibrillary acidic protein. The expression of Thy 1 mRNA was reported in untreated RGC-5 cells before [9], which is consistent with the original report by Krishnamoorthy et al. [4]. We also observed an increase after 72 h of incubation. However, neural markers like tau, β-III tubulin, MAP-1b, MAP2 and PGP9.5 were found [14]. On the other hand, Ganapathy et al. used staurosporine to differentiate RGC-5 cells, and they postulated that the RGC-5 cells developed a more neuronal phenotype upon staurosporine treatment. These findings suggest that progressive sub-culturing of cell lines alters gene expression, as previously reported [47,48,49].

### 3.2. The Reaction of 661W Cells to TSA Incubation

The untreated 661W cells shared several similarities with RGC-5 cells, like a highly comparable morphology. Apart from this, distinct differences between the 661W and RGC-5 cells could be detected under TSA incubation. The 661W cells reacted more sensitively to the cytotoxic effects of TSA, especially under long-term incubation. The loss of 661W cells was more distinct, and the viability of those cells dropped under 50%, while the RGC-5 cells doubled their metabolic activity after 120 h of incubation. In contrast with the RGC-5 cells, the 661W cells did not increase their expression of β-III tubulin significantly after long-term incubation, nor did they show a significant relative rise in Thy-1-mRNA in qRT-PCR when incubated with TSA, but they showed an unexpected increase in the PGP9.5 mRNA. On the other hand, we observed a significant increase in CRX mRNA when using Wood’s protocol, which in turn indicates a photoreceptor cell line. In general, it can be stated that TSA has a toxic effect on 661W cells, whereas the RGC-5 cells showed signs of differentiation.

In the literature, very little is known about the influence of TSA on 661W cells or cone photoreceptor cells in general. Chen et al. investigated the effect of HDAC inhibitors on retinal cells and found that they inhibited the differentiation of photoreceptors [50], and another publication described the TSA-mediated induction of apoptosis via upregulation of Apaf-1 in 661W cells [51]. Our findings confirm this rather toxic effect on 661W cells.

### 3.3. The Effects of TSA

HDAC inhibitors like TSA are mainly involved in regulating gene expression in post-mitotic cells. Aside from histones, they have many other targets like transcription factors, which they either activate or inactivate. In this manner, they manipulate the phenotypes of cells [52,53,54]. In cancer therapy, HDAC inhibitors are an interesting target as they operate differently on mitotic than on post-mitotic cells [55,56]. In transformed cells, HDAC inhibitors lead to terminal differentiation and induce mitotic arrest. In contrast, differentiated cells are relatively resistant to HDAC inhibitor-induced cell death [57,58,59]. Especially in the field of neurosciences, HDAC inhibitors are known to induce terminal differentiation in precursor cells [31,32].

TSA is also known to stop the cell cycle, but the detailed mechanisms are only partially understood. For example, TSA causes depletion of cyclin D1, a cofactor of different transcription factors, which leads to cell cycle arrest [60,61,62], to name one possible pathway. Hence, it might be possible that TSA, in the case of RGC-5 cells, first leads to cell cycle arrest and afterward to a switch in the expression profile toward a neuronal or even ganglion cell-like phenotype. This could be one possible explanation for the circumstance that neuronal differentiation in RGC-5 cells was mostly seen after long-term TSA incubation.

### 3.4. The Problem of Cell Line Contamination

Aside from all the benefits of using cell lines to study new therapies or possible physiological conditions in vitro instead of investigating native tissues or animal models, there is always the risk of contamination. Today, at least 15–20% of all cell lines used in research are supposedly contaminated or wrongly categorized [63]. This is why more and more researchers call for action and demand stricter rules in cell line research [64,65]. Contamination of cell lines could be detected since the establishment of genetic markers in 1967 [66], and short tandem repeat profiling is a safe validation method [67,68].

In summary, the RGC-5 cell line analyzed here is murine. It is unclear at what point in the generation of the rat cell line the contamination with mouse cells occurred. Therefore, the real origin is unknown. According to all analyses, the two cell lines presented clear differences, which could mean that they are not of the same murine origin or background or that they developed quite differently over time.

As the true nature of the RGC-5 cell line is mostly unsure, and its origin cannot clearly be trailed back even in the literature [21,41,69,70,71], one cannot transfer results out of RGC-5 experiments to the real ganglion cell physiology anymore. In addition, one also cannot transfer results from the RGC-5 experiments to the 661W cell line or photoreceptor properties. Although a few researchers still use these cells [28,29], several journals, including *IOVS*, *Experimental Eye Research* and *Molecular Vision*, stopped publishing articles based on RGC-5-based experiments in the context of ganglion cell research [70,72,73]. There are now several successful approaches to culturing RGC, and although this is much more complex, these cells reflect the true phenotype of neuronal cells [74,75,76].

### 3.5. Contributions to Knowledge

The study undertaken confirmed the murine origin of both the 661W and RGC-5 cell lines. To examine their characteristics, standardized redifferentiation protocols were employed, revealing distinct morphological and neuronal marker properties and suggesting they are not identical or at least possess significantly different traits.

Notably, the RGC-5 cells displayed a higher expression of the neuronal marker β-III tubulin and a relative increase in Thy-1 mRNA compared with the 661W cells, indicating a more neuron-like nature. These findings shed light on their divergent properties.

Moreover, the study underscored the importance of regular standardized tests to uphold the quality of cell lines utilized in research. It emphasized the potential risks of contamination and loss of cell properties when working with cell lines like RGC-5 and 661W.

## 4. Materials and Methods

### 4.1. Cell Culture

Professor Neeraj Agarwal (UNT Health Science Centre, Fort Worth, TX, USA) provided RGC-5 cells (passage 5). The mouse photoreceptor cell line 661W (passage 78) originated from Prof. Dr. Muayyad Al-Ubaidi. Through all experiences, both cell lines were maintained in Dulbecco’s Modified Eagle’s Medium (Gibco, Thermo Fisher Scientific, Karlsruhe, Germany, supplemented with 2 nM L-glutamin, 100 U/L penicillin G, 1 µg/mL streptomycin sulfate and 0–10% fetal bovine serum (Thermo Fisher Scientific, Karlsruhe, Germany), according to the utilized protocol, at 37 °C in an incubator at 5% CO_2_. They were seeded at a density of 175,000 cells/well in 6-well plate, a density of 60,000 cells/well in 24-well plate and a density of 10,000 cells/well in a 96-well plate. Afterward, the RGC-5 cells and 661W cells were treated for 24, 48, 72, 96 and 120 h with 500 nM TSA according to two published protocols [14,16] to cause redifferentiation. Phase contrast pictures were taken from the 24-well cultures (100× magnification) with an Axiovert 135 microscope (Zeiss, Göttingen, Germany) which used AxioVision 4.6 software.

### 4.2. Chromosome Analysis

The culturing and harvesting of the cells (P6 of RGC-5 and P80 of 661W) followed a standard protocol [77]. After GTG banding, numerical analysis of 11 cells and structural analysis of 7 cells in each case were performed.

### 4.3. SDS-PAGE and Western Blot

Cells were cultured with TSA added to the medium over 24, 48, 72, 96 and 120 h, and protein was collected by using a cell extraction buffer (Invitrogen, Thermo Fisher Scientific, Karlsruhe, Germany) with PMSF and protease inhibitor (Merck, Darmstadt, Germany). The protein concentration was determined using a BCA Protein Assay Kit (Applichem, Darmstadt, Germany). Equal amounts of protein (10 µg) were loaded onto 12% SDS gels. After electrophoresis, a wet transfer to a nitrocellulose membrane was performed, where the membrane was blocked in 5% BSA. Immunostaining was conducted using antibodies against caspase 3, Bax, Bcl-2 and β-III tubulin as well as one against actin for normalization. All antibodies were purchased from Cell Signaling in Germany and diluted to a 1:1000 ratio. Chemoluminescence was detected by the ECL chemoluminescence system (Thermo Fisher Scientific, MA, USA) transforming the marked membranes into X-ray films. The protein levels were quantified based on densitometry, using ImageJ software Version 1.51 on the scanned films three times for each membrane. The BAX, Bcl-2, caspase and β-III tubulin levels were normalized to the actin levels and a control.

### 4.4. Immunofluorescence Staining

Following TSA incubation for 24, 48, 72, 96 and 120 h, the cells were fixated with 4% paraformaldehyde, permeabilized with 0.1% Triton-X-100, blocked with 4% BSA and incubated over night with the primary antibody at 4 °C. The primary antibodies targeted human NF (goat antibody diluted to 1:100, R&D Systems, Minneapolis, MN, USA), β-III tubulin (mouse antibody diluted to 1:100, R&D Systems, USA), MAP 2 (rabbit antibody diluted to 1:250, Millipore, Germany) and the Tau protein (mouse antibody diluted to 1:100, Merck, Darmstadt, Germany). The next day, after washing, the cells were incubated at room temperature for 1 h with the corresponding secondary antibody (diluted to 1:1000) linked to a fluorescence dye (Alexa flour 488, Invitrogen, Germany or Cy3, Jackson ImmunoResearch Europe, Ely, UK). All this was followed by 4’,6-diamidino-2-phenylindole (DAPI) staining. Images of this fluorescence were taken at 100× magnification from the 24-well plate. Controls without the primary antibodies were performed with every staining procedure to verify whether the secondary antibody showed unspecific attachment. None of them showed any undefined staining.

### 4.5. qRT-PCR

The cells were collected after 24 and 120 h of TSA treatment, and mRNA was isolated from the cells and reverse transcribed using a MultiMACS cDNA Synthesis Kit (Miltenyi Biotec, Bergisch Gladbach, Germany) on the MultiMACS™ M96 Separator (Miltenyi Biotec, Bergisch Gladbach, Germany) according to the manufacturer’s protocol. After cDNA synthesis, quantitative real-time PCR was performed over 45 cycles using the Universal SYBER Green Supermix for PCR (Biorad, Hercules, CA, USA) on a thermocycler (Eppendorf, Hamburg, Germany). The cDNA expression levels of the investigated genes, Thy-1 and GFAP were normalized to the cDNA level of the housekeeping gene L32.

Conventional PCR products of Thy-1, PGP9.5, GFAP and L32 were obtained with the primers designed for real-time PCR, for Thy-1 FOR 5′-AGCCAGATGCCTGAAAGAGA-3′ and REV 5′- GGGCTGAGAATGACCTGGTA -3′, for CRX FOR 5′TATATGAACCCGGGACCTCA 3′ and CRX REV 5′CCTCACGTGCATACACATCC 3′, for PGP9.5 FOR5′-CCCTTGGTTTGCAGCTTTAG-3′ and REV 5′-CACATCCAAGGCCGTAACTT-3′, for GFAP for 5′-TTAGTGTACCCTCTCGGAAG-3′ and REV 5′-AGGTTAGCAGAGGTGACAAG-3′ and for L32 for 5′-AACCGAAAAGCCATCGTAGAA-3′ and REV 5′-CCTGGCGTTGGGATTGG-3′. Since the origin of the cells was unclear, all primers were designed to fit 100% to both the rat and mouse genomes. The analysis of data was performed using Biorad software (BioRadCFXManager 2.0). To evaluate the relative expression of the target gene’s mRNA in relation to the control, Pfaffl’s formula [78] was applied.

### 4.6. MTS Viability Assay

After 24, 48, 72, 96 and 120 h of supplementation with TSA, 20 µL of CellTiter 96^®^ AQueous One Solution Reagent (Promega, Madison, WI, USA) was directly added to each culture well (96-well plate). The RGC-5 and 661W cells were incubated for 90 min, and absorbance was recorded at 490 nm with a microplate reader (BioTek, Synergy HT, Germany) with the correction of interference at 690 nm. Each experiment was conducted six times (n = 6).

### 4.7. Crystal Violet Staining

Following the MTS assay, the cells were fixed with 4% paraformaldehyde overnight. After three washing steps, they were stained with crystal violet solution (Sigma Alderich, Steinheim, Germany) and then washed again three times. Next, they were incubated with 1% SDS for 1 h. Absorbance was measured at 595 nm. Each experiment was conducted six times (n = 6).

### 4.8. Caspase 3/7 Activity Assay

After incubation with TSA, the caspase 3/7 activity was determined by using a CaspaseGlo 3/7 activity kit (Promega, Madison, WI, USA) according to the manufacturer’s protocol, where 100 µL of the CaspaseGlo 3/7 reagent was added directly to each well (96-well plate). After 60 min of incubation at room temperature, the luminescence was measured with a luminometer (BioTek, Synergy HT, Germany). Each experiment was conducted six times (n = 6).

### 4.9. Statistical Analysis

Data are represented as the mean and SD. Statistical analysis was performed using JMP^®^ (version 11.0.0, SAS Institute Inc., Cary, NC, USA). ANOVA with a Tukey–Kramer or Dunett’s post hoc test was used to validate the differences between the different testing groups and between the treated cells and the control. Differences were considered significant at *p* < 0.05.

## 5. Conclusions

The analysis of the 661W and RGC-5 cell lines revealed valuable insights into their molecular properties and origin. Both cell lines were confirmed to be of murine origin. While some experimental methods yielded similar results for both cell lines, the majority of the methods showed distinct differences. Notably, the RGC-5 cells exhibited higher expression of the neuronal marker β-III tubulin, a relative increase in Thy-1 mRNA and distinct effects of TSA on CRX mRNA, indicating significant disparities between the two cell lines. Additionally, karyotyping clearly demonstrated differences in the genotypes of the 661W and RGC-5 cells, further supporting their distinct identities. Based on these findings, it is evident that 661W and RGC-5 cells are not interchangeable, and results from one cell line cannot be extrapolated to the other. Consequently, for the integrity of scientific research, the use of the RGC-5 cell line should be discontinued. This study underscores the importance of standardized quality control measures even for seemingly well-characterized cell lines, emphasizing the need to implement uniform guidelines for cell line authentication and characterization in research. In the future, researchers will need to exercise caution in the selection and use of cell lines and ensure their authenticity and reliability. Incorporating the latest technologies and various learning methods, such as advanced genomics and cell imaging techniques, can further improve the accuracy and reproducibility of research results.

## Figures and Tables

**Table 1:** The dispute about the origin of RGC-5 cells and possible contamination with 661W cells.

Protocol	Schwechter et al. [16]	Wood et al. [14]
**Morphology**	**RGC-5:** Differentiation toward a neuronal phenotype after long-term incubation with TSA. **661W:** Fewer cells changed morphologies, and neurites were fewer and shorter. Importantly, the majority of cells died during long-term incubation.	**Both:** Similar trend to that in Schwechter et al.
**β-III tubulin**	**RGC-5:** In Western blot analysis as well as in the immunohistochemistry, maximum values for long-term incubation (96 h and 120 h) were with TSA. **661W:** First, an increase in β-III tubulin expression could be observed. After 72 h, values dropped again.	**RGC-5:** Maximum increase could be detected after long-term incubation. Increase in expression was stronger than in treatment after Schwechter protocol. **661W:** No increase in β-III tubulin expression under long-term incubation.
**MAP-2, Tau**	**Both:** Expression increased with long-term incubation.	**Both:** Expression increased.
**Thy-1 mRNA**	**Both:** No effect	**RGC-5:** Slight increase after incubation time of 120 h. **661W:** No effect
**PGP9.5 mRNA**	**Both:** No effect	**661W:** Twofold increase in mRNA expression after 120 h.
**CRX mRNA**	**RGC-5:** Sevenfold increase in mRNA expression after 120 h. **661W:** No effect	**RGC-5:** Slight decrease in mRNA expression after 120 h. **661W:** Fourfold increase after 120 h.

## Data Availability

Data is contained within the article.

## References

[1] Stitt A.W., Curtis T.M., Chen M., Medina R.J., McKay G.J., Jenkins A., Gardiner T.A., Lyons T.J., Hammes H.P., Simo R. (2016). The progress in understanding and treatment of diabetic retinopathy. Prog. Retin. Eye Res..

[2] United States Environmental Protection Agency (2019). Directive to Prioritize Efforts to Reduce Animal Testing.

[3] Schnichels S., Paquet-Durand F., Loscher M., Tsai T., Hurst J., Joachim S.C., Klettner A. (2021). Retina in a dish: Cell cultures, retinal explants and animal models for common diseases of the retina. Prog. Retin. Eye Res..

[4] Krishnamoorthy R.R., Agarwal P., Prasanna G., Vopat K., Lambert W., Sheedlo H.J., Pang I.H., Shade D., Wordinger R.J., Yorio T. (2001). Characterization of a transformed rat retinal ganglion cell line. Brain Res. Mol. Brain Res..

[5] Agarwal N., Agarwal R., Kumar D.M., Ondricek A., Clark A.F., Wordinger R.J., Pang I.H. (2007). Comparison of expression profile of neurotrophins and their receptors in primary and transformed rat retinal ganglion cells. Mol. Vis..

[6] Nieto P.S., Acosta-Rodriguez V.A., Valdez D.J., Guido M.E. (2010). Differential responses of the mammalian retinal ganglion cell line RGC-5 to physiological stimuli and trophic factors. Neurochem. Int..

[7] Aoun P., Simpkins J.W., Agarwal N. (2003). Role of PPAR-gamma ligands in neuroprotection against glutamate-induced cytotoxicity in retinal ganglion cells. Investig. Ophthalmol. Vis. Sci..

[8] Fan W., Agarwal N., Cooper N.G. (2006). The role of CaMKII in BDNF-mediated neuroprotection of retinal ganglion cells (RGC-5). Brain Res..

[9] Ganapathy P.S., Dun Y., Ha Y., Duplantier J., Allen J.B., Farooq A., Bozard B.R., Smith S.B. (2010). Sensitivity of staurosporine-induced differentiated RGC-5 cells to homocysteine. Curr. Eye Res..

[10] Harper M.M., Adamson L., Blits B., Bunge M.B., Grozdanic S.D., Sakaguchi D.S. (2009). Brain-derived neurotrophic factor released from engineered mesenchymal stem cells attenuates glutamate- and hydrogen peroxide-mediated death of staurosporine-differentiated RGC-5 cells. Exp. Eye Res..

[11] Maher P., Hanneken A. (2005). Flavonoids protect retinal ganglion cells from oxidative stress-induced death. Investig. Ophthalmol. Vis. Sci..

[12] Maher P., Hanneken A. (2005). The molecular basis of oxidative stress-induced cell death in an immortalized retinal ganglion cell line. Investig. Ophthalmol. Vis. Sci..

[13] Van Bergen N.J., Wood J.P., Chidlow G., Trounce I.A., Casson R.J., Ju W.K., Weinreb R.N., Crowston J.G. (2009). Recharacterization of the RGC-5 retinal ganglion cell line. Investig. Ophthalmol. Vis. Sci..

[14] Wood J.P., Chidlow G., Tran T., Crowston J.G., Casson R.J. (2010). A comparison of differentiation protocols for RGC-5 cells. Investig. Ophthalmol. Vis. Sci..

[15] Schnichels S., Schultheiss M., Hofmann J., Szurman P., Bartz-Schmidt K.U., Spitzer M.S. (2012). Trichostatin A induces cell death at the concentration recommended to differentiate the RGC-5 cell line. Neurochem. Int..

[16] Schwechter B.R., Millet L.E., Levin L.A. (2007). Histone deacetylase inhibition-mediated differentiation of RGC-5 cells and interaction with survival. Investig. Ophthalmol. Vis. Sci..

[17] Frassetto L.J., Schlieve C.R., Lieven C.J., Utter A.A., Jones M.V., Agarwal N., Levin L.A. (2006). Kinase-dependent differentiation of a retinal ganglion cell precursor. Investig. Ophthalmol. Vis. Sci..

[18] Harvey R., Chintala S.K. (2007). Inhibition of plasminogen activators attenuates the death of differentiated retinal ganglion cells and stabilizes their neurite network in vitro. Investig. Ophthalmol. Vis. Sci..

[19] Lieven C.J., Millet L.E., Hoegger M.J., Levin L.A. (2007). Induction of axon and dendrite formation during early RGC-5 cell differentiation. Exp. Eye Res..

[20] Schultheiss M., Schnichels S., Miteva K., Warstat K., Szurman P., Spitzer M.S., Van Linthout S. (2012). Staurosporine-induced differentiation of the RGC-5 cell line leads to apoptosis and cell death at the lowest differentiating concentration. Graefes Arch. Clin. Exp. Ophthalmol..

[21] Krishnamoorthy R.R., Clark A.F., Daudt D., Vishwanatha J.K., Yorio T. (2013). A Forensic Path to RGC-5 Cell Line Identification: Lessons Learned. Investig. Ophthalmol. Vis. Sci..

[22] Kanan Y., Hoffhines A., Rauhauser A., Murray A., Al-Ubaidi M.R. (2009). Protein tyrosine-O-sulfation in the retina. Exp. Eye Res..

[23] Kanan Y., Moiseyev G., Agarwal N., Ma J.X., Al-Ubaidi M.R. (2007). Light induces programmed cell death by activating multiple independent proteases in a cone photoreceptor cell line. Investig. Ophthalmol. Vis. Sci..

[24] Schnichels S., Hagemann U., Januschowski K., Hofmann J., Bartz-Schmidt K.U., Szurman P., Spitzer M.S., Aisenbrey S. (2013). Comparative toxicity and proliferation testing of aflibercept, bevacizumab and ranibizumab on different ocular cells. Br. J. Ophthalmol..

[25] Tsuruma K., Yamauchi M., Inokuchi Y., Sugitani S., Shimazawa M., Hara H. (2012). Role of oxidative stress in retinal photoreceptor cell death in N-methyl-N-nitrosourea-treated mice. J. Pharmacol. Sci..

[26] Kolb H., Kolb H., Fernandez E., Nelson R. (1995). Morphology and Circuitry of Ganglion Cells. Webvision: The Organization of the Retina and Visual System.

[27] Greeff R. (1900). Mikroskopische Anatomie des Sehnerven und der Netzhaut. Handbuch der gesamten Augenheilkunde: I.

[28] Mu L., Dong Z., Zhang Y. (2022). Mechanisms of Qing-Gan Li-Shui Formulation in Ameliorating Primary Open Angle Glaucoma: An Analysis Based on Network Pharmacology. Evid. Based Complement. Alternat. Med..

[29] Duan C.Y., Fan W.L., Chen F. (2023). Roles of Optineurin and Extracellular Vesicles in Glaucomatous Retinal Cell Loss. Curr. Med. Sci..

[30] Xi X., Ma J., Chen Q., Wang X., Xia Y., Wen X., Yuan J., Li Y. (2022). Acteoside attenuates hydrogen peroxide-induced injury of retinal ganglion cells via the CASC2/miR-155/mTOR axis. Ann. Transl. Med..

[31] Gaub P., Tedeschi A., Puttagunta R., Nguyen T., Schmandke A., Di Giovanni S. (2010). HDAC inhibition promotes neuronal outgrowth and counteracts growth cone collapse through CBP/p300 and P/CAF-dependent p53 acetylation. Cell Death Differ..

[32] Kretsovali A., Hadjimichael C., Charmpilas N. (2012). Histone deacetylase inhibitors in cell pluripotency, differentiation, and reprogramming. Stem Cells Int..

[33] Olney J.W. (1969). Glutaate-induced retinal degeneration in neonatal mice. Electron microscopy of the acutely evolving lesion. J. Neuropathol. Exp. Neurol..

[34] Vorwerk C.K., Lipton S.A., Zurakowski D., Hyman B.T., Sabel B.A., Dreyer E.B. (1996). Chronic low-dose glutamate is toxic to retinal ganglion cells. Toxicity blocked by memantine. Investig. Ophthalmol. Vis. Sci..

[35] Sippl C., Bosserhoff A.K., Fischer D., Tamm E.R. (2011). Depletion of optineurin in RGC-5 cells derived from retinal neurons causes apoptosis and reduces the secretion of neurotrophins. Exp. Eye Res..

[36] Ju W.K., Liu Q., Kim K.Y., Crowston J.G., Lindsey J.D., Agarwal N., Ellisman M.H., Perkins G.A., Weinreb R.N. (2007). Elevated hydrostatic pressure triggers mitochondrial fission and decreases cellular ATP in differentiated RGC-5 cells. Investig. Ophthalmol. Vis. Sci..

[37] Wood J.P., Lascaratos G., Bron A.J., Osborne N.N. (2007). The influence of visible light exposure on cultured RGC-5 cells. Mol. Vis..

[38] Beale R., Osborne N.N. (1982). Localization of the Thy-1 antigen to the surfaces of rat retinal ganglion cells. Neurochem. Int..

[39] Xiang M., Zhou L., Macke J.P., Yoshioka T., Hendry S.H., Eddy R.L., Shows T.B., Nathans J. (1995). The Brn-3 family of POU-domain factors: Primary structure, binding specificity, and expression in subsets of retinal ganglion cells and somatosensory neurons. J. Neurosci..

[40] Nieto P.S., Valdez D.J., Acosta-Rodriguez V.A., Guido M.E. (2011). Expression of novel opsins and intrinsic light responses in the mammalian retinal ganglion cell line RGC-5. Presence of OPN5 in the rat retina. PLoS ONE.

[41] Al-Ubaidi M.R. (2014). RGC-5: Are they really 661W? The saga continues. Exp. Eye Res..

[42] Thompson A.F., Crowe M.E., Lieven C.J., Levin L.A. (2015). Induction of Neuronal Morphology in the 661W Cone Photoreceptor Cell Line with Staurosporine. PLoS ONE.

[43] Thompson A.F., Levin L.A. (2010). Neuronal differentiation by analogs of staurosporine. Neurochem. Int..

[44] Furukawa T., Morrow E.M., Cepko C.L. (1997). Crx, a novel otx-like homeobox gene, shows photoreceptor-specific expression and regulates photoreceptor differentiation. Cell.

[45] Chen S., Wang Q.L., Nie Z., Sun H., Lennon G., Copeland N.G., Gilbert D.J., Jenkins N.A., Zack D.J. (1997). Crx, a novel Otx-like paired-homeodomain protein, binds to and transactivates photoreceptor cell-specific genes. Neuron.

[46] Rath M.F., Morin F., Shi Q., Klein D.C., Moller M. (2007). Ontogenetic expression of the Otx2 and Crx homeobox genes in the retina of the rat. Exp. Eye Res..

[47] Fourrier M.C., Arnold M.F., Collet B., Munro E.S. (2009). The effect of sub-culturing on the basal level of type I interferon (IFN) gene expression in the Salmon Head Kidney (SHK-1) cell line. Fish Shellfish Immunol..

[48] Muff R., Rath P., Ram Kumar R.M., Husmann K., Born W., Baudis M., Fuchs B. (2015). Genomic instability of osteosarcoma cell lines in culture: Impact on the prediction of metastasis relevant genes. PLoS ONE.

[49] AbuHammad S., Zihlif M. (2013). Gene expression alterations in doxorubicin resistant MCF7 breast cancer cell line. Genomics.

[50] Chen B., Cepko C.L. (2007). Requirement of histone deacetylase activity for the expression of critical photoreceptor genes. BMC Dev. Biol..

[51] Wallace D.M., Cotter T.G. (2009). Histone deacetylase activity in conjunction with E2F-1 and p53 regulates Apaf-1 expression in 661W cells and the retina. J. Neurosci. Res..

[52] Glozak M.A., Sengupta N., Zhang X., Seto E. (2005). Acetylation and deacetylation of non-histone proteins. Gene.

[53] Quivy V., Van Lint C. (2004). Regulation at multiple levels of NF-kappaB-mediated transactivation by protein acetylation. Biochem. Pharmacol..

[54] Ryu H., Lee J., Olofsson B.A., Mwidau A., Dedeoglu A., Escudero M., Flemington E., Azizkhan-Clifford J., Ferrante R.J., Ratan R.R. (2003). Histone deacetylase inhibitors prevent oxidative neuronal death independent of expanded polyglutamine repeats via an Sp1-dependent pathway. Proc. Natl. Acad. Sci. USA.

[55] Hrabeta J., Stiborova M., Adam V., Kizek R., Eckschlager T. (2014). Histone deacetylase inhibitors in cancer therapy. A review. Biomed. Pap. Med. Fac. Univ. Palacky Olomouc Czechoslov..

[56] Xu W.S., Parmigiani R.B., Marks P.A. (2007). Histone deacetylase inhibitors: Molecular mechanisms of action. Oncogene.

[57] Burgess A., Ruefli A., Beamish H., Warrener R., Saunders N., Johnstone R., Gabrielli B. (2004). Histone deacetylase inhibitors specifically kill nonproliferating tumour cells. Oncogene.

[58] Insinga A., Monestiroli S., Ronzoni S., Gelmetti V., Marchesi F., Viale A., Altucci L., Nervi C., Minucci S., Pelicci P.G. (2005). Inhibitors of histone deacetylases induce tumor-selective apoptosis through activation of the death receptor pathway. Nat. Med..

[59] Ungerstedt J.S., Sowa Y., Xu W.S., Shao Y., Dokmanovic M., Perez G., Ngo L., Holmgren A., Jiang X., Marks P.A. (2005). Role of thioredoxin in the response of normal and transformed cells to histone deacetylase inhibitors. Proc. Natl. Acad. Sci. USA.

[60] Alao J.P., Gamble S.C., Stavropoulou A.V., Pomeranz K.M., Lam E.W., Coombes R.C., Vigushin D.M. (2006). The cyclin D1 proto-oncogene is sequestered in the cytoplasm of mammalian cancer cell lines. Mol. Cancer.

[61] Alao J.P., Lam E.W., Ali S., Buluwela L., Bordogna W., Lockey P., Varshochi R., Stavropoulou A.V., Coombes R.C., Vigushin D.M. (2004). Histone deacetylase inhibitor trichostatin A represses estrogen receptor alpha-dependent transcription and promotes proteasomal degradation of cyclin D1 in human breast carcinoma cell lines. Clin. Cancer Res..

[62] Zhang Z.K., Davies K.P., Allen J., Zhu L., Pestell R.G., Zagzag D., Kalpana G.V. (2002). Cell cycle arrest and repression of cyclin D1 transcription by INI1/hSNF5. Mol. Cell. Biol..

[63] MacLeod R.A., Dirks W.G., Matsuo Y., Kaufmann M., Milch H., Drexler H.G. (1999). Widespread intraspecies cross-contamination of human tumor cell lines arising at source. Int. J. Cancer.

[64] Nardone R.M. (2008). Curbing rampant cross-contamination and misidentification of cell lines. BioTechniques.

[65] Nardone R.M. (2007). Eradication of cross-contaminated cell lines: A call for action. Cell Biol. Toxicol..

[66] Masters J.R., Thomson J.A., Daly-Burns B., Reid Y.A., Dirks W.G., Packer P., Toji L.H., Ohno T., Tanabe H., Arlett C.F. (2001). Short tandem repeat profiling provides an international reference standard for human cell lines. Proc. Natl. Acad. Sci. USA.

[67] Barallon R., Bauer S.R., Butler J., Capes-Davis A., Dirks W.G., Elmore E., Furtado M., Kline M.C., Kohara A., Los G.V. (2010). Recommendation of short tandem repeat profiling for authenticating human cell lines, stem cells, and tissues. Vitr. Cell. Dev. Biol. Anim..

[68] Eltonsy N., Gabisi V., Li X., Russe K.B., Mills G.B., Stemke-Hale K. (2012). Detection algorithm for the validation of human cell lines. Int. J. Cancer.

[69] Agarwal N. (2013). RGC-5 cells. Investig. Ophthalmol. Vis. Sci..

[70] Clark A., Tamm E.R., Al-Ubaidi M.R., Hollyfield J.G. (2013). On the use of immortalized ocular cell lines in vision research: The unfortunate story of RGC-5. Exp. Eye Res..

[71] Sippl C., Tamm E.R. (2014). What is the nature of the RGC-5 cell line?. Adv. Exp. Med. Biol..

[72] Hao M., Liu Y., Chen P., Jiang H., Kuang H.Y. (2018). Astragaloside IV protects RGC-5 cells against oxidative stress. Neural Regen. Res..

[73] Wei Z., Mingxing W., Shanxue L., Chao L., Yanqiu Z., Duoduo X.U., Jian W. (2022). Buyang Huanwu Tang protects HO-induced RGC-5 cell against oxidative stress and apoptosis reactive oxygen species-mitogen-activated protein kinase signaling pathway. J. Tradit. Chin. Med..

[74] Park Y.H., Snook J.D., Zhuang I., Shen G., Frankfort B.J. (2020). Optimized culture of retinal ganglion cells and amacrine cells from adult mice. PLoS ONE.

[75] Luo Z., Chang K.C., Wu S., Sun C., Xia X., Nahmou M., Bian M., Wen R.R., Zhu Y., Shah S. (2022). Directly induced human retinal ganglion cells mimic fetal RGCs and are neuroprotective after transplantation in vivo. Stem Cell Rep..

[76] Castillo B., del Cerro M., Breakefield X.O., Frim D.M., Barnstable C.J., Dean D.O., Bohn M.C. (1994). Retinal ganglion cell survival is promoted by genetically modified astrocytes designed to secrete brain-derived neurotrophic factor (BDNF). Brain Res..

[77] Barch M.J.K.T., Spurbeck J.L. (1997). The AGT Cytogenetics Laboratory Manual.

[78] Pfaffl M.W. (2001). A new mathematical model for relative quantification in real-time RT-PCR. Nucleic Acids Res..

